# Transcription Profiling of Epstein-Barr Virus Nuclear Antigen (EBNA)-1 Expressing Cells Suggests Targeting of Chromatin Remodeling Complexes

**DOI:** 10.1371/journal.pone.0012052

**Published:** 2010-08-10

**Authors:** Ramakrishna Sompallae, Simone Callegari, Siamak Akbari Kamranvar, Maria G. Masucci

**Affiliations:** Department of Cell and Molecular Biology, Karolinska Institutet, Stockholm, Sweden; Hannover Medical School, Germany

## Abstract

The Epstein-Barr virus (EBV) encoded nuclear antigen (EBNA)-1 regulates virus replication and transcription, and participates in the remodeling of the cellular environment that accompanies EBV induced B-cell immortalization and malignant transformation. The putative cellular targets of these effects of EBNA-1 are largely unknown. To address this issue we have profiled the transcriptional changes induced by short- and long-term expression of EBNA-1 in the EBV negative B-cell lymphoma BJAB. Three hundred and nineteen cellular genes were regulated in a conditional transfectant shortly after EBNA-1 induction while a ten fold higher number of genes was regulated upon continuous EBNA-1 expression. Promoter analysis of the differentially regulated genes demonstrated a significant enrichment of putative EBNA-1 binding sites suggesting that EBNA-1 may directly influence the transcription of a subset of genes. Gene ontology analysis of forty seven genes that were consistently regulated independently on the time of EBNA-1 expression revealed an unexpected enrichment of genes involved in the maintenance of chromatin architecture. The interaction network of the affected gene products suggests that EBNA-1 may promote a broad rearrangement of the cellular transcription landscape by altering the expression of key components of chromatin remodeling complexes.

## Introduction

Epstein-Barr virus (EBV) is a gamma herpes virus that infects the majority of human adults. Like other herpes viruses, EBV has a dual life cycle characterized by the establishment of latency in B-lymphocytes and productive infection in epithelial cells [1], [2]. Latently infected B-cells express a restricted set of viral genes that promote a global rearrangement of the cellular environment, leading to B-cell growth transformation and immortalization [2], [3]. Reprogramming of cell proliferation and apoptosis and deregulation of genome integrity maintenance machineries are likely to underlie the association of EBV with a variety of malignancies, including virtually all cases of endemic Burkitt's lymphoma (BL) and nasopharyngeal carcinoma (NPC) and approximately half of Hodgkin's lymphomas (HL) [1], [4]. These proliferating cells express distinct subsets of latency genes that define viral programs, known as latency I, II and III, whose common denominator is the regular expression of the EBV nuclear antigen (EBNA)-1.

EBNA-1 is required for the correct partitioning of viral episomes during cell division [5], [6] and regulates the activity of viral promoters. Hence, binding of EBNA-1 to the family-of-repeats (FR) locus in the latent origin of replication, *oriP*, positively regulates the expression of the Cp promoter that drives the transcription of six EBNA genes in cells expressing latency III [7], [8] while binding to specific sites in the Qp promoter is involved in the autoregulation of EBNA-1 expression in different latency types [9]. EBNA-1 binds also to cellular DNA, although with a lower affinity compared to viral DNA [10]. A set of EBNA-1 binding sites was recently characterized in the human genome using chromatin immunoprecipitation and a microarray of human promoter sequences [11]. Surprisingly several promoters containing EBNA-1 binding sites were not regulated by EBNA-1 in luciferase reporter assays [12] suggesting that additional transcription factors, or the chromatin architecture in which transcription occurs, may influence the effect of EBNA-1. Thus, while the capacity of EBNA-1 to act as a transcription regulator may contribute to reshape the cellular environment during EBV infection and malignant transformation, the primary and secondary targets of this effect remain unknown.

Gene expression profiling has been used to dissect the influence of EBNA-1 on cellular transcription. By mining public gene expression databases of EBV positive B-cell lines expressing different types of latency we have identified the gene encoding for the catalytic subunit of the NADPH oxidase NOX2 as a transcriptional target of EBNA-1 whose upregulation may be involved in the induction of DNA damage and genomic instability in lymphoid malignancies [13]. Stable or transient transfection of EBNA-1 was shown to affect the expression of a range of cellular genes in NPC and HD models, including genes involved in transcription, translation and cell signaling [14], [15]. Validation of a subset of the regulated genes revealed that EBNA-1 enhances the activity of transcription factors, including STAT1, AP1, c-Jun and ATF2 and regulates a variety of cellular processes including TGF-β signaling, cytokine production and angiogenesis [15], [16]. The mechanisms by which EBNA-1 exerts such pleyotropic effect on a wide variety of diverse cellular processes remains, however, unclear.

We have addressed this issue by comparing the transcription profiles of stable and conditional EBNA-1 transfected sublines of the EBV negative B-cell lymphoma BJAB where EBNA-1 was expressed for few days, few months or several years. Our data suggest that, while exerting a direct effect on a subset of the regulated genes, EBNA-1 may orchestrate a broad rearrangement of transcription by regulating the expression of genes involved in nucleosome maintenance and chromatin architecture.

## Materials and Methods

### Experimental design

In order to examine the effect of EBNA-1 on the expression of cellular genes, transcription microarray profiling was performed in transfected sublines of the EBV negative B-lymphoma line BJAB that express EBNA-1 either stably [17] or under the control of a tetracycline regulated promoter [13]. Short-term effects were monitored within two weeks of EBNA-1 upregulation in BJAB-tTAE1 while long term effects were monitored in cells that continuously expressed EBNA-1 for few months, BJAB-tTAE1, or for several years, BJAB-E1. Non-specific effects were accounted by comparing the gene expression profiles of untransfected cells or cells transfected with the empty vector (BJAB-E1/BJAB and BJAB-tTAE1/BJAB-tTA pairs) or cultured in the presence or absence of doxycyline (BJAB-tTAE1 +/− Dox and BJAB-tTA +/− Dox pairs). Expression profiles were collected from three independent experiments where each EBNA-1 +/− pair was tested in parallel (Table 1).

**Table 1:** Experimental design and RNA labeling.

Experiment group	Sample cell line (Cy5)	Control cell line (Cy3)
Stable expression	BJAB-E1	BJAB
Inducible short-term expression	BJAB-tTA-E1 (−Tet)	BJAB-tTA-E1 (+Tet)
Inducible long-term expression	BJAB-tTA-E1 (−Tet)	BJAB-tTA (−Tet)
Tetracycline treatment	BJAB-tTA (−Tet)	BJAB-tTA (+Tet)

RNA prepared from cells expressing EBNA-1 was labeled with Cy-5, while RNA from control cells with Cy-3. Labeled RNA was then co-hybridized on to individual microarray to capture the transcriptional changes induced in EBNA-1 expressing cells.

### Cell cultures and RNA extraction

The cells were grown in RPMI-1640 complete medium supplemented with penicillin (100 U/ml), streptomycin (0.1 mg/ml), 10% fetal calf serum and the appropriate selection drugs for BJAB-E1 (200 mg/ml geneticin - Gibco, Darmstadt, Germany) or BJAB-tTA/BJAB-tTAE1 (0.5 µg/ml hygromycin - Calbiochem, San Diego, CA, USA and 0.5 µg/ml puromycin - Sigma-Aldrich, St. Louis, MO, USA). One mg/ml of doxycycline was added to the BJAB-tTAE1 culture medium to repress the expression of EBNA-1. Three aliquots of 10^7^ cells were collected from each cell line and culture condition, and EBNA-1 expression was monitored in Western blots using the OT1x mouse monoclonal antibody as described [13]. RNA extraction was performed with the RNeasy kit (QIAGEN®) according to the recommended protocol. On-column DNase-I digestion was performed to ensure the elimination of genomic DNA and the quality of the RNA was controlled by agarose gel electrophoresis before preparation for microarray analysis.

### Microarray analysis

Gene expression profiling was performed at the Core Facility of the Institute of Biochemistry, Hannover Medical School, Hannover, Germany, using oligonucleotide-based Agilent G4112F 44K HD arrays (design ID 014850) as recommended by the manufacturer (Two-Color Microarray-Based Gene Expression Analysis V5.0.1; Agilent Technologies). RNA preparations were transcribed into Cy3-labelled (control EBNA-1 negative cells) or Cy5-labelled (EBNA-1 expressing cells) cRNA, and hybridized pair wise onto the microarray (Table 1). Data extraction was performed using the Agilent feature extraction software V9.1.3.1. The output files from each microarray include the signal (MedianSignal), standard deviation of the signal (PixSDev), background (BGMedianSignal), and corrected signal (ProcessedSignal -dye-normalized signal after surrogate algorithm, used for computation of ratio values) for each channel. From these, a signal-to-noise ratio for each channel (signal/background) and relative error (standard deviation of signal/signal) were calculated. Differences in the expression levels of individual genes were expressed as ratios reporting the change in intensity between cells with EBNA-1 (Cy5) and control (Cy3) channels. Ratios and quality features were calculated using the software Genedata Expressionist Pro (Genedata AG, Basel, Switzerland). The data has been deposited in NCBI gene expression data, under accession number GSE22964.

### Quality control and data analysis

The ratio values and quality information including signal-to-noise ratio and relative error values of each probe were used for further analysis. Gene filtering and selection of differentially regulated genes was done using the R package (http://www.r-project.org). Qualified probes were selected with a signal-to-noise ratio higher than 2, and relative error lower than 0.25. Hierarchical clustering was performed to assess the reliability of the gene expression data in classifying the groups. Differentially regulated probes were extracted using as cut-off ratio values≥1.25 and ≤0.75 corresponding to 25% change in mRNA level. Samples derived from three independent experiments were analysed separately and only genes showing concordant regulation in each of the experiments were considered as differentially regulated.

### Quantitative Real-Time Polymerase Chain Reaction (qPCR)

The transcription levels of a selected pool of differentially regulated genes were verified by qPCR. RNA was extracted using TRIzol reagent and reverse transcription was performed as described [13]. A reaction mix (20µl) containing 20 ng cDNA, 500 nM of the forward and reverse primers for each gene (Table S1), Kapa SybrFast qPCR Master Mix (10µl, Kapa Biossystems, Woburn, USA) and water was added to a Fast Optical 96 well plate (Applied Biosystems, Foster City, USA) and placed into a 7000 Sequence Detection Systems thermocycler (Applied Biosystems). The samples were heated to 50°C for 2 min followed by 95°C for 10 min and 40 cycles of 95°C for 15 sec and 60°C for 1 min were conducted. The signals were analyzed using the ABI PRISM 7000 software (Applied Biosystems) and the fold increase was calculated using the ΔΔct method. The GAPDH levels were similar in parental and stable transfected cell lines (ct value<0.5). Melting curves were used to assess the specificity of the primers. In each case, the presence of amplified fragments of the expected size and absence of non-specific products was confirmed by agarose gel electrophoresis of the PCR products (data not shown).

### Promoter analysis

Promoter analysis was carried out to identify putative transcriptional targets of EBNA-1. Promoter sequences, identified as the 5′ flanking sequence between 3 kb upstream to 0.5 kb downstream of the transcription start site of all human genes, were obtained from the Ensembl database (http://www.ensembl.org/ release 53). To predict possible EBNA-1 binding sites sequence patterns and hidden Markov model (HMM) profiles were generated using 13 non-redundant sequences from the EBV genome. These included known EBNA-1 binding sites in the family of repeats (FR) and dyad symmetry (DS) elements of oriP [5], EBNA-1 binding sites in the Qp promoter [9] and Rep* site [18]. The derived sequence pattern RRTWRBVYRYRYTDYY (IUPAC nomenclature where R is purine, Y is pyrimidine W is A or T, V is not T, D is not C and B is not A) was then used to search the human promoter database. HMMs were constructed using the HMMER suite of programs to create a statistical profile of EBNA-1 binding sequences [19]. The HMM training set was enlarged to include, in addition to the viral promoters, 60 human promoter sequences that were found to contain EBNA-1 binding sites as assessed by chromatin immunoprecipitation (ChIP, 27 promoters) or both ChIP and electrophoretic mobility shift analysis (EMSA, 33 promoters) [12]. The HMM results were further categorized based on E-values and raw scores and the score of the Rep*1 site was used as cut-off. Enrichment of putative EBNA-1 binding elements in the promoters of the regulated genes was assessed based on the random occurrence of such elements in the whole genome. A list of 958 known transcription regulators (TRs) was obtained from the gene ontology (GO) category “transcription regulator activity” (GO Id:0030528).

### Analysis of Gene ontology and protein-protein interaction networks

The GO categories of genes whose expression was differentially regulated were identified using the Database for Annotation, Visualization and Integrated Discovery (DAVID) [20]. The GO categories with default P-value≤0.1 were considered as significantly affected [21]. Protein-protein interaction networks were generated using selected sets of genes from the enriched GO categories and high confidence interaction data were collected by diverse experimental methods available at the human protein reference database (HPRD) [22]. The generated network modules were visualized in the software environment Cytoscape [23].

## Results

### Gene expression profile of EBNA-1 expressing cells

The effect of EBNA-1 on the expression of cellular genes was investigated by transcription profiling of transfected sublines of the EBV negative B-lymphoma BJAB. In order to discriminate between early and late EBNA-1 effects, the analysis was performed in the conditional BJAB-tTAE1 cell line within two weeks of doxycycline withdrawal, when the expression of EBNA-1 had reached plateau levels, and after several months of culture in doxycycline free medium. The latter pattern of gene expression was compared to that of a stable BJAB-E1 cell line that was kept in culture for several years. EBNA-1 specific effects were in every case assessed by comparison with the appropriate EBNA-1 negative control and non-specific effects caused by doxycycline treatment were accounted for by excluding genes that were regulated in vector transfected BJAB-tTA. In order to increase the statistical significance all pair-wise comparisons were performed in triplicate and only genes showing ≥25% change in each of the triplicates were considered as differentially regulated.

**Figure 1:**
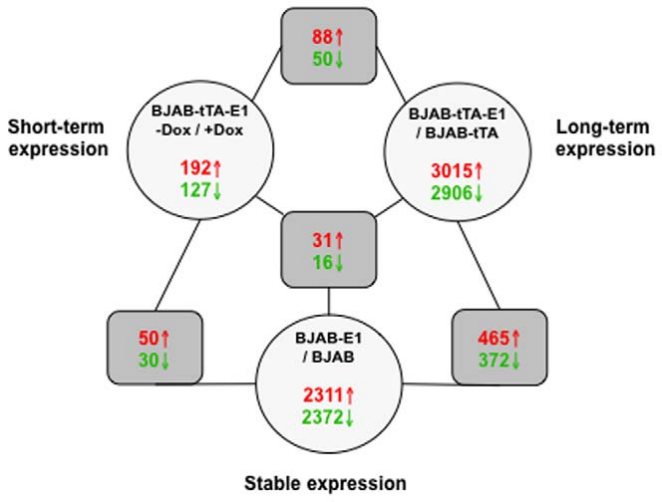
Summary of differentially regulated genes in EBNA-1 expressing cells. The numbers of differentially regulated genes in short-term, long-term and stable EBNA-1 expressing cells are shown. Arrows indicate up (**↑**) and downregulated (**↓**) genes with ≥1.25 fold change in each of the triplicate experiments. Forty-seven genes are commonly regulated independently on the time of EBNA-1 expression.

An overview of the genes regulated in different conditions of EBNA-1 expression is shown in Figure 1. A relatively small group of 319 genes, 192 upregulated and 127 downregulated, were specifically affected by short-term expression of EBNA-1 in BJAB-tTAE1 while the number of affected genes was increased by approximately 10 fold upon long term EBNA-1 expression (5921 genes of which 3015 upregulated and 2906 downregulated) and in the stable transfectant BJAB-E1 (4383 genes of which 2311 upregulated and 2372 downregulated). Approximately 15% of the genes regulated upon long-term and stable EBNA-1 expression were affected in both conditions (837 genes of which 465 upregulated and 372 downregulated). Only 47 genes, 31 upregulated and 16 downregulated, were consistently affected independently on the time of EBNA-1 expression. A list of these commonly regulated genes is shown in Table S2. Analysis of the chromosomal distribution of the differentially regulated transcripts showed that the altered genes are widely spread on all chromosomes resulting in a global rearrangement of the transcription landscape (Figure S1).

### Promoter analysis of the regulated genes

**Figure 2:**
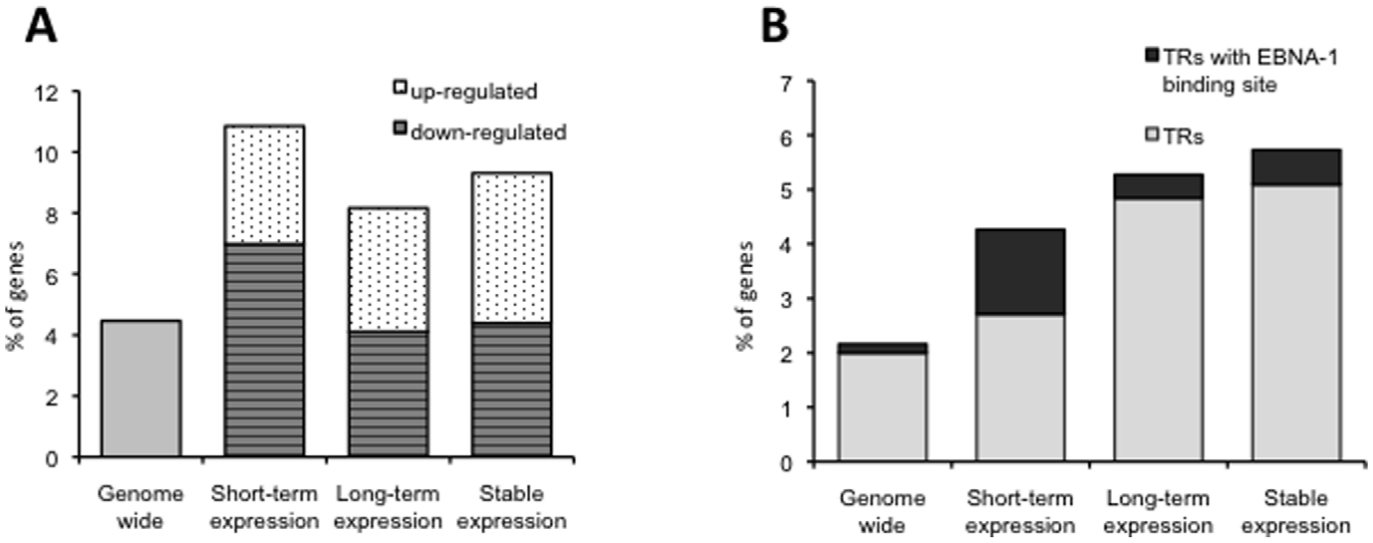
Promoter analysis of differentially expressed genes and genes encoding transcription regulators. **A**) Histogram showing the percentage of genes that contain putative EBNA-1 binding sites in their promoters. More than 2-fold enrichment of promoters containing putative EBNA-1 binding elements was observed in EBNA-1 regulated genes compared to the genome wide random frequency. 28 regulated genes (10 upregulated and 18 downregulated) in short-term expressing cells contain one or more putative EBNA-1 binding sites in their promoters, while upon long term and stable EBNA-1 expression EBNA-1 binding site was detected in 359 (181 upregulated and 178 downregulated) and 307 (162 upregulated and 145 downregulated) regulated genes respectively. B) Percentages of TRs encoded in the human genome and differentially expressed in the presence of EBNA-1. Several TRs containing putative EBNA-1 binding sites in their promoter sequences were regulated in EBNA-1 expressing cells. The enrichment was particularly prominent after short-term EBNA-1 expression.

EBNA-1 binds to cellular DNA and was shown to regulate the transcription of viral and cellular promoters [7], [8], [13]. In order to assess whether binding to regulatory DNA sequences may be involved in the transcriptional effect of EBNA-1, putative promoter regions located −3000 to +500 bp relative to the transcription start of regulated genes were searched for the presence of putative EBNA-1 binding sites using an HMM profile generated from known EBNA-1 binding sequences. Putative EBNA-1 binding sites were identified in 4.5% of promoter regions across the human genome while a significant 2-fold enrichment was observed among the genes differentially regulated under all conditions of EBNA-1 expression (Figure 2A). Two or more binding sites were found in 10–20% of these promoters and four promoters in this group were previously shown to interact with EBNA-1 in ChIP-chip assays [12] (Table S3).

To further assess whether the transcriptional effect of EBNA-1 may be broadened through the activation of cellular transcription regulators we looked for this functional category amongst the EBNA-1 regulated genes. Genes annotated in the Gene Ontology (GO) database as “Transcription regulators” (TR) constitute 2.2% of the human genome and 7.5% of them have promoters containing putative EBNA-1 binding sites (72 out of 958 gene). TRs were significantly enriched among the regulated genes in all conditions of EBNA-1 expression (11, 232 and 182 TRs differentially regulated in short-term, long-term and stably EBNA-1 expressing cells, corresponding to 2, 2.4 and 2.6 fold enrichment, respectively) (Figure 2B). One or more EBNA-1 binding sites were present in the promoters of 4 out of 11 TRs (36.4%) that were regulated early affected by EBNA-1 induction (Table S4).

### Gene Ontology analysis of the differentially expressed genes

Given the large discrepancy in the number of genes regulated upon short- and long-term EBNA-1 expression, we focused our analysis on the 47 genes that were regularly affected independently of the time of expression (Figure 1 and Table S2). The most upregulated gene in this group was the putative transmembrane phospholipid-transporter ATPase Class II type 9A (ATP9A) and consistently upregulated were also the tumor necrosis factor receptor-associated factor 1 (TRAF1), that was earlier shown to be upregulated in EBV infected cells [24], [25]. Ten out of 31 upregulated transcripts were histone H2B variants encoded in different gene clusters (Table S2). The most downregulated gene was Membrane Metallo-Endopeptidase (MME), also known as common acute lymphocytic leukemia antigen, CALLA, CD10 or NEP, that is highly expressed in mature germinal center B-cells. Previous studies have shown that downregulation of MME imparts a growth advantage to various cancers [26].

The GO annotation of the regulated genes was then used to gain insights on the biological functions that may be modulated by EBNA-1. This analysis revealed a significant enrichment of GO terms belonging to the biological processes “Maintenance of chromatin” and “Regulation of apoptosis” (p-value≤0.1, Table 2). The enrichment in the “Regulation of Apoptosis” category is in line with earlier reports on the anti-apoptotic property of the EBV latency I gene expression program [27]. Under “Molecular function” the commonly affected genes were found in categories associated with nucleotide binding and phosphatase activity, while the “Cellular component” category showed enrichment of genes encoding for proteins associated with chromosomes. The protein products of the genes regulated in the “Maintenance of chromatin” category were histone H2B variants, all upregulated, and the DNA binding proteins SWItch/Sucrose NonFermentable (SWI/SNF) related, matrix associated, actin dependent regulator of chromatin, subfamily b, member 1 (SMARCB1, also known as hSNF5) and immunoglobulin μ binding protein 2 (IGHMBP2), both downregulated. In the “Regulation of Apoptosis” category were, in addition to TRAF1, non-metastatic cells protein expressed 5 (NME5), protein C (PROC), and galectin-1 (LGALS1), all upregulated. NME5 belongs to a family of nucleoside diphosphate kinases (NDKs) that is overexpressed in cancers [28] and was reported to play a major role in promoting cell survival in a mouse model [29]. PROC and galectin-1 encode a vitamin K-dependent serine protease and a β-galactoside-binding protein that modulate cell-cell and cell-matrix interactions [30], [31]. Additional interacting and catalytic partners of the genes enriched in “Biological process” were found in the “Molecular function” category. The ATPases, ATP8B2, ATP9A2, and microtubule associated serine/threonine kinase family member 4 (MAST4) were upregulated while protein kinase AKT2 (also known as PRKBB) and Sodium/potassium-transporting ATPase gamma chain (FXYD2) were downregulated. In addition to the genes identified in the “Chromatin maintenance” category, the “Chromosome” category enriched under “Cellular component” included NCAPH2, the H2 regulatory subunit of the non-SMC condensin II complex [32] that was downregulated.

**Table 2:** GO categories that are significantly affected in all conditions of EBNA1 expression.

Biological Process	Level	No. of genes	*P*	Up-regulated	Down-regulated
**Cellular component organization and biogenesis**					
Establishment and/or maintenance of chromatin architecture	5	12	<0.001	HIST1H2BJ, HIST1H2BN, HIST1H2BM, HIST3H2BB, HIST1H2BD, HIST1H2BO, HIST1H2BC, HIST1H2BG, HIST1H2BI, HIST1H2BL	SMARCB1, IGHMBP2
**Regulation of cellular process**					
Regulation of apoptosis	5	4	0.100	NME5, LGALS1, PROC, TRAF1	
**Molecular function**					
**Binding**					
Adenyl nucleotide binding	4	6	0.092	NME5, ATP8B2, ATP9A, MAST4	AKT2, IGHMBP2,
**Catalytic activity**					
Nucleoside-triphosphatase activity	7	4	0.093	ATP8B2, ATP9A	FXYD2, IGHMBP2
**Cellular component**					
**Non-membrane bound organelle**					
Chromosome	5	12	<0.001	HIST1H2BJ, HIST1H2BN, HIST1H2BM, HIST3H2BB, HIST1H2BD, HIST1H2BO, HIST1H2BC, HIST1H2BG, HIST1H2BI, HIST1H2BL	SMARCB1, NCAPH2

**Figure 3:**
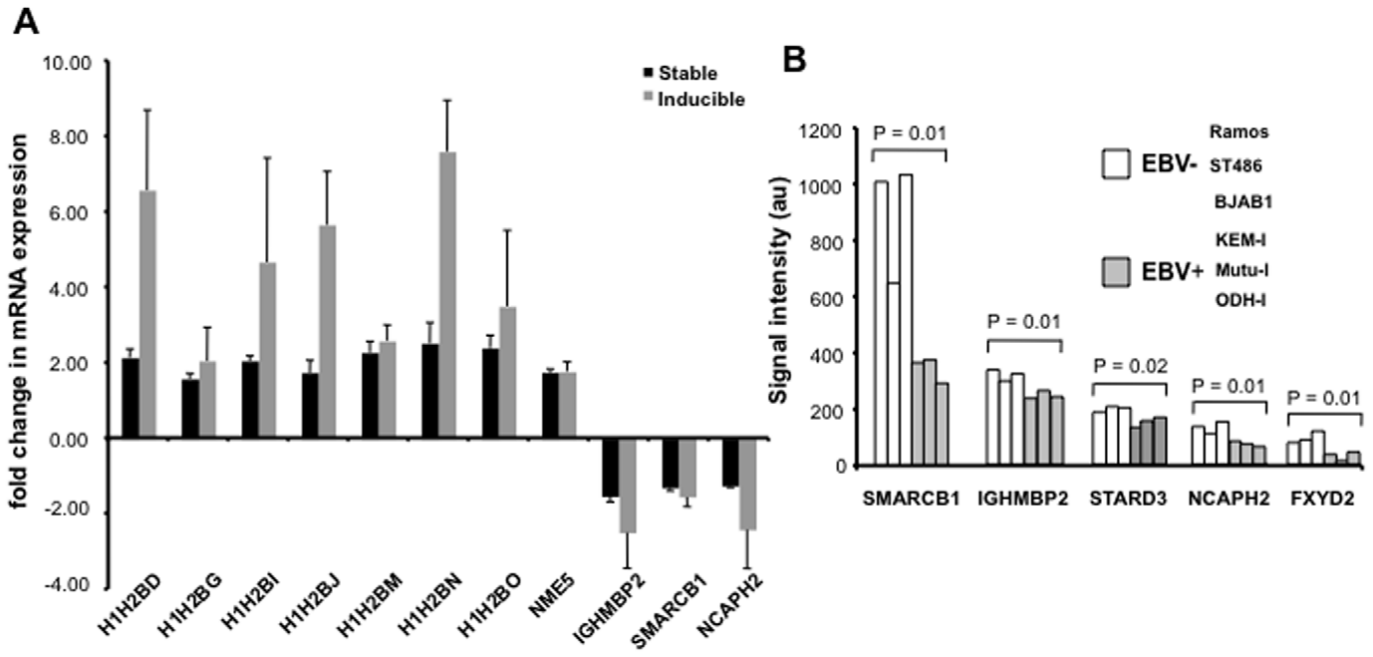
qPCR based validation of selected genes. qPCR analysis was performed on selected differentially expressed genes **A**). Relative expression (fold change) of selected genes in inducible and stable EBNA-1 expressing cells compared to controls. The means and standard error of three independent experiments are shown. B) Genes regulated in EBV positive BLs expressing latency I compared EBV negative BLs. Signal intensities (au = arbitrary units) obtained from the normalized data. A significant decrease in the mRNA levels of SMARCB1, NCAPH2, IGHMBP2, STARD3 and FXYD2 is observed in the cells expressing EBV latency type I compared to EBV negative BLs.

The unexpected enrichment of genes involved in the regulation of chromatin maintenance prompted us to investigate whether the effect could be confirmed by conventional qPCR analysis. The mRNA levels of seven histone H2B variants, SMARCB1, IGHMBP2, NCAPH2, and NME5 were tested in triplicate samples of RNA isolated from paired BJAB/BJAB-E1 and BJAB-tTAE1 cells cultured with or without doxycycline. A good correlation between the microarray and qPCR data was found for all the genes tested (Figure 3A), confirming that these genes are regulated shortly after EBNA-1 expression. In order to assess the general validity of this observation we turned to publically available gene expression microarray datasets of EBV negative BL lines and EBV carrying cell lines expressing latency I where EBNA-1 is the only detected viral protein (NCBI gene expression data, GSE2350). Thirty-eight probes corresponding to 27 of the 47 commonly regulated genes (Table S2) were present in these microarrays. Five of the 27 genes: SMARCB1, IGHMBP2, NCAPH2, STARD3 and FXYD2 were consistently downregulated in BLs expressing latency I (KEM-I, Mutu-I and ODH-I) compared to EBV negative B-lymphoma lines (Ramos, ST486 and BJAB) (Figure 3B). The consistent downregulation of SMARCB1, IGHMBP2 and NCAPH2 in both EBNA-1 transfectants and EBNA-1 expressing BLs strengthen the conclusion that the effect of EBNA-1 on transcription is likely to involve the activity of chromatin remodeling complexes.

### Protein-protein interaction network analysis

**Figure 4:**
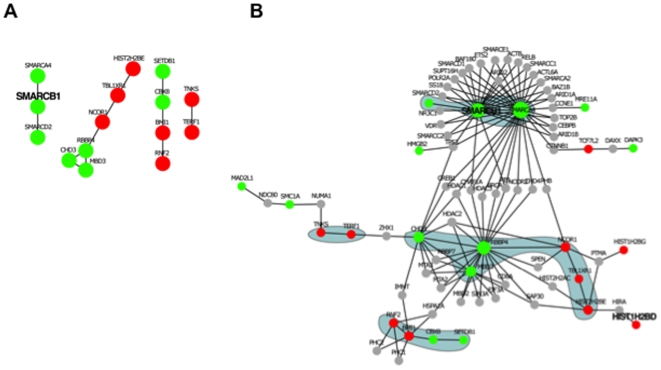
Protein interaction networks involving genes with chromatin maintenance function. Networks were derived from the high confidence protein interaction data of chromatin maintenance genes that were regulated upon long-term EBNA-1 expression. A) Four protein-interaction sub-networks identified three chromatin-remodeling complexes, SWI/SNF, NuRD and PRC1, that are downregulated and one telomere associated complex that is upregulated. Upregulated genes are indicated in red and downregulated genes are in green. Genes that are regulated in both short- and long-term EBNA-1 expression are indicated in bold. B) Network analysis shows that the SWI/SNF and NuRD complexes interact with key enzymes required for chromatin remodeling such HDACs. Sub-networks are identified by shaded areas. The size of the nodes is proportional to the number of interactions.

Comparison of the GO categories of the commonly regulated genes versus genes regulated in short-term, long-term and stable EBNA-1 expressing cells revealed a progressive expansion of the number of affected genes in each category depending on the duration of EBNA-1 expression (Table 3). To assess whether this expansion involves also chromatin remodeling complexes, genes annotated in the “Chromatin maintenance” category were identified in the long-term and stable EBNA-1 expression datasets and protein interaction data were extracted for the products of 43 genes that were found in both datasets (Table S5). This analysis revealed the presence of four protein interaction sub-networks (Figure 4A). One sub-network included, in addition to SMARCB1, two additional subunits of the SWI/SNF complex, SMARCA4 and SMARCD2 [33]. The second sub-network contained the CHD3, MBD3 and RBBP4 subunits of the Nucleosome Remodeling and Deacetylase (NuRD) complex [33], [34] and three known interacting partners. The third cluster was formed by the CBX8, BMI1 and RNF2 (RING1B) subunits of the Polycomb repressive complex-1 (PRC1) [35] and their interacting partner SETDB1. The fourth sub-network included the telomere associated proteins TNKS and TERF1 [36]. The three chromatin-remodeling complexes identified by this analysis are involved in transcriptional repression [33], [34], [37]. All the affected subunits of the SWI/SNF and NuRD complexes were downregulated in EBNA-1 expressing cells while E3 ubiquitin ligases BMI1 and RNF2 of the PRC1 complex were upregulated. However, the nucleosome binding subunit CBX8, that anchors the complex to methylated histone H3K27 through its chromo domain [38], was downregulated suggesting that also the PRC1 complex may be functionally inactivated in EBNA-1 expressing cells.

**Table 3:** Expansion of selected GO categories during prolonged EBNA1 expression.

GO category	GO Level	Common		Short-term induced		Long-term induced		Stable	
Biological process		No. of genes	*P*	No. of genes	*P*	No. of genes	*P*	No. of genes	*P*
**Cellular component organization and biogenesis**	2	14	0.062	67	<0.001	774	<0.001	622	<0.001
Establishment and/or maintenance of chromatin architecture	5	12	<0.001	24	<0.001	144	<0.001	93	0.001
**Regulation of cellular process**									
Regulation of apoptosis	5	4	0.100	14	0.020	146	0.003	137	<0.001
**Molecular function**									
**Binding**	2	32	0.023	184	0.001	3017	<0.001	2544	<0.001
Adenyl nucleotide binding	4	6	0.092	26	0.094	438	<0.001	363	<0.001
**Catalytic activity**									
Nucleoside-triphosphatase activity	6	4	0.094	16	0.011	157	0.009	134	0.020
**Cellular component**									
**Non-membrane-bound organelle**	2	13	0.029	40	0.075	510	<0.001	403	0.019
Chromosome	5	12	<0.001	21	0.001	151	<0.001	110	<0.001

To further explore the observation that chromatin-remodeling complexes are affected by EBNA-1 expression, we generated a highly connected protein network by expanding the sub-networks with their unregulated protein partners. Only unregulated nodes (proteins) that are connected to two or more regulated partners were kept to generate a network where all the end nodes are differentially regulated (Figure 4B). This analysis revealed that the chromatin remodeling complexes that are regulated by EBNA-1 expression form hubs of a protein interaction network. Thus, subunits of both the SWI/SNF and NuRD complexes interact with Histone deacetylases (HDACs) that are required for transcription repression. Downregulation of both the SWI/SNF and NuRD complexes is therefore likely to concur in promoting transcription by preventing the recruitment of HDACs.

## Discussion

EBNA-1 is expressed in all EBV-associated malignancies and may play a role in cell transformation and tumorigenesis [39], [40]. Since EBNA-1 does not exert known enzymatic activities, these effects are likely to be mediated by its well-documented capacity to regulate transcription [14], [15]. Indeed, EBNA-1 binds to both viral [41], [42] and cellular promoters [12] and modulates the activity of cellular transcription factors [16], but the mechanisms by which the viral protein reshapes the transcriptional landscape and the critical cellular targets are largely unknown. We have addressed this issue by comparing the whole-genome transcription profiles of cell lines expressing stable or inducible EBNA-1 on the assumption that common features of the early and late transcriptional signatures might identify regulatory nodes that could explain the pleyotropic consequences of EBNA-1 expression.

Our data confirm the previously reported broad effect of stable or long-term EBNA-1 expression on cellular transcription (Figure 1). One possible mechanism of this effect is the capacity of EBNA-1 to directly influence the transcription of at least a subset of the regulated genes. In support of this possibility, we have found a statistically significant two fold enrichment in EBNA-1 binding sites by scanning the promoters of the regulated genes with an HMM profile constructed from known EBNA-1 binding sequences (Figure 2A). The failure to demonstrate enrichment of EBNA-1 binding sites in the promoters of genes regulated by expression of a dominant negative EBNA-1 in the 721 LCL, reported by Dresang et al. using a similar search algorythm [12], is likely to be explained by a confounding effect on the transcription of other EBV latency genes that are also expressed in LCL cells. Further support to the possibility of a direct effect of EBNA1 on transcription comes from the observation that known EBNA-1 transcriptional targets, such as FR and Qp promoter in the EBV genome, contain at least two EBNA-1 binding sites [42], [43] suggesting that this may be a distinctive feature of the regulated promoters. Our HMM based algorithm identified regulated genes with promoters containing multiple EBNA-1 binding in both short- and long-term EBNA-1 expressing cells (Table S3) and the validity of this prediction is confirmed by the finding that EBNA-1 binds to at least four of the predicted sequences in ChIP-on-chip assays [12]. However, while these observations strengthen the assumption of a direct correlation between the binding of EBNA-1 to cellular promoter sequences and the transcriptional response, the mechanism this regulation remains unclear. In particular, the failure of EBNA-1 to regulate the activity of several cellular promoters containing binding sites in luciferase reporter assays [12] indicates that, when present, the transcriptional effect is complex and probably dependent on co-factors and on the chromatin context in which transcription occurs. It is also important to stress that, in addition to interacting with cellular DNA in regions homologous to the viral DNA binding, EBNA-1 binds to the small groove of cellular DNA through Gly-Arg-rich domain that resemble the AT-hook motifs of High Mobility Group A (HMGA) proteins [44]. HMGA proteins are architectural transcription factors that regulate the activity of a variety of genes. While lack direct transcriptional activation capacity, these proteins regulate gene expression by changing the conformation of DNA and/or by direct interaction with several transcription factors (reviewed in [45]). It remains to be seen whether the AT-hook domain of EBNA-1 could play a role in the regulation of transcriptional.

In a possible scenario, the transcriptional effect of EBNA-1 could be amplified through the regulation of cellular transcription factors. Consistent with this possibility, a more than 2-fold enrichment of genes annotated as transcription regulators (TRs), including both transcription factors and components of the transcription machinery, was observed in all conditions of EBNA-1 expression. This finding is in line with previously documented capacity of EBNA-1 to activate transcription factors such as STAT1 and AP-1 [15], [16]. Interestingly, TRs containing EBNA-1 binding sites in their promoters were particularly enriched among the genes regulated in short-term EBNA-1 expressing cells (Figure 2B). These included for example STAT4, a member of the STAT family of transcription factors that regulates cytokine responses and lymphocyte differentiation [46].

While the large number of genes affected in long-term and stable EBNA-1 expressing cells corroborates the notion that EBNA-1 activates of a broad transcriptional network, a novel aspect of this regulation was revealed by the analysis of genes that are affected shortly after induction of EBNA-1 in the conditional BJAB transfectant. Only 47 genes were regularly affected in both short- and long-term EBNA-1 expressing cells suggesting that their products may be critically required for the phenotypic effects of EBNA-1. GO analysis of these commonly regulated transcripts revealed an unexpected enrichment in genes involved in the maintenance of chromatin architecture. These included several variants of histone H2B encoded in different gene clusters. Interestingly, increased protein levels of several of H2B variants were detected by quantitative mass spectrometry in short-term EBNA-1 expressing cells (Sompallae et al. unpublished observation). However, simple analysis Western blot did not revels significant changes in the steady state levels of the proteins suggesting that the turnover of H2B variants may be increased. Indeed, it was recently shown that H2B that is not incorporated into core histones is highly toxic for the cell and is rapidly eliminated by proteasome dependent degradation resulting in virtually unchanged steady state levels of the proteins [47]. Histone H2B plays a major role in the regulation of transcription via tightly orchestrated cycles of ubiquitination and deubiquitination [48]. The deubiquitinating enzyme USP7 that binds with high affinity to EBNA-1 was recently shown to deubiquitinate histone H2B and contribute thereby to the transcriptional activity of EBNA-1 [49]. Recent findings suggest that excess H2B that is not incorporated into core histones is highly toxic for the cell and is rapidly eliminated by proteasome dependent degradation resulting in virtually unchanged steady state levels of the proteins.

Several subunits of nucleosome organizing complexes were also present among the commonly regulated genes. Nucleosomes constitute a potent obstacle for biological processes requiring access to DNA, such as transcription, DNA replication and repair. The cellular machinery allowing the access of transcription factors to their target promoters includes histone acetyltransferases (HATs) and deacetylases (HDACs) and several chromatin-remodeling complexes. HATs add acetyl groups to the amino-terminal tails of histones weakening the interaction with DNA or the neighboring nucleosomes. HDACs facilitate the compaction of DNA by reversing histone acetylation while chromatin-remodeling complexes use the energy of ATP hydrolysis to weaken the interaction between histone core particles and DNA. At least three chromatin-remodeling complexes were consistently affected in EBNA-1 expressing cells. SMARCB1, a core component of all variants of the SWI/SNF ATPase chromatin remodeling complexes [50], [51], was consistently downregulated (Figure 3A). A 2-fold decrease of SMARCB1 mRNA was also revealed by the comparison of transcription profiles of EBV negative B-cell lines and BL lines expressing latency I collected in public databases (Figure 3B). Hence, SMARCB1 downregulation may be a regular consequence of EBNA-1 expression. SMARCB1 acts as a tumor suppressor whose mutations or loss were shown to cause malignant rhabdoid tumors, an extremely aggressive cancer of early childhood [52], [53] and accumulation of DNA damage [54]. Two additional subunits of the SWI/SNF complex, SMARCA4 and SMARCD2, were downregulated in long-term EBNA-1 expressing cells, suggesting that the complex may be progressively inactivated (Figure 4A). Several subunits of the NuRD and PRC1 complexes were also downregulated. The PRC1 complex mediates the ubiquitination of histones via its E3 ligase subunits [35], while, similar to SWI/SNF, the NuRD complex uses the energy of ATP to remodel nucleosome structures via interaction with HDAC enzymes (Figure 4B).

Collectively, our findings indicate that at least three mechanisms could contribute to a broad rearrangement of the transcription landscape in EBNA-1 expressing cells. Direct regulation of a subset of cellular promoters containing putative EBNA-1 binding sites and direct or indirect effects on several cellular transcription factors may initiate a transcription cascade that is greatly amplified upon prolonged EBNA-1 expression. In addition, an epigenetic reorganization of the cellular transcriptional milieu may occur shortly after EBNA-1 expression through the regulation of several molecular complexes that control chromatin architecture. This could in turn open the access to transcription factors and promote the wide-ranging effects on gene expression induced by EBNA-1.

## Supporting Information

Figure S1 Distribution of EBNA-1 regulated genes on chromosomes. The chromosomal distribution of genes regulated on short-term, long-term and stable EBNA-1 expressing conditions. The up- and downregulated genes on each chromosome and fold change are represented by x- and y-axis respectively. All genes with fold change greater than 10 were scaled to the fixed value of 10 in the plots.
(1.56 MB TIF)

Table S1 Primers used for Q-PCR analysis.
(0.04 MB DOC)

Table S2 List of genes that are regularly affected independently on the time of EBNA-1 expression.
(0.08 MB DOC)

Table S3 List of differentially regulated genes that contain multiple putative EBNA-1 binding sites in their promoter.
(0.12 MB DOC)

Table S4 List of differentially regulated TRs and contain EBNA-1 binding sites in their promoter.
(0.07 MB DOC)

Table S5 List of regulated genes included in protein interaction analysis.
(0.08 MB DOC)
