# Supplementary figures and images for: Transcription Profiling of Epstein-Barr Virus Nuclear Antigen (EBNA)-1 Expressing Cells Suggests Targeting of Chromatin Remodeling Complexes
Source: PLoS One. 2010 Aug 10;5(8):e12052. doi: 10.1371/journal.pone.0012052 (PMC2919392; doi:10.1371/journal.pone.0012052)

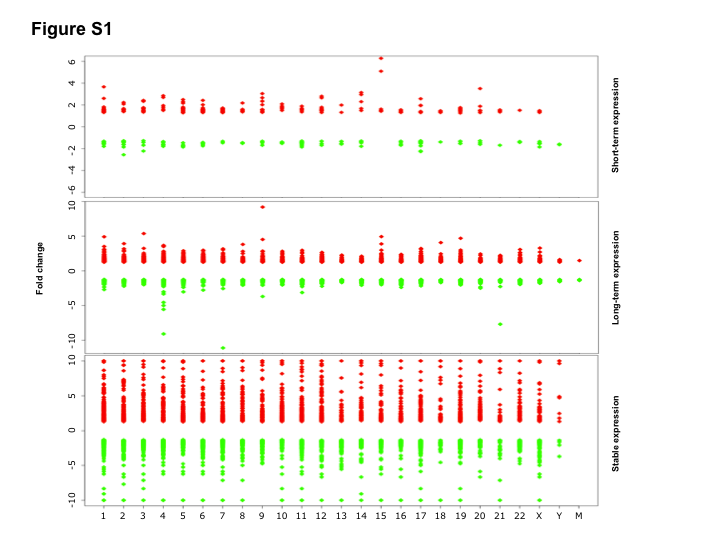

Supplement: Figure S1 — Distribution of EBNA-1 regulated genes on chromosomes. The chromosomal distribution of genes regulated on short-term, long-term and stable EBNA-1 expressing conditions. The up- and downregulated genes on each chromosome and fold change are represented by x- and y-axis respectively. All genes with fold change greater than 10 were scaled to the fixed value of 10 in the plots. (1.56 MB TIF) [file pone.0012052.s001.tif]
