# Supplementary material for: Transcription Profiling of Epstein-Barr Virus Nuclear Antigen (EBNA)-1 Expressing Cells Suggests Targeting of Chromatin Remodeling Complexes
Source: PLoS One. 2010 Aug 10;5(8):e12052. doi: 10.1371/journal.pone.0012052 (PMC2919392; doi:10.1371/journal.pone.0012052)
Supplement: Table S1 — Primers used for Q-PCR analysis. (0.04 MB DOC) [file pone.0012052.s002.doc]

**Table S1.** Primers used for Q-PCR analysis.

| **Gene** | **Sense primer (5’- 3’)** | **Anti-sense primer (5’- 3’)** | **Product (bp)** |
| --- | --- | --- | --- |
| *HIST1H2BD* | CAGTGTTCTAACTATTAACGCTACG | GACCTGCTTCAGCACCTTGT | 172 |
| *HIST1H2BG* | CTGGCGCATTACAACAAGC | GAGTTTTAAAGCACCTAAGCACAC | 167 |
| *HIST1H2BI* | CTGGCGCATTACAACAAGC | TCGTTAGCGCTTTTACCCAG | 170 |
| *HIST1H2BJ* | CTGGCGCATTACAACAAGC | GAGAGTTTGCAACCAACTCACT | 165 |
| *HIST1H2BM* | CTGGCGCATTACAACAAGC | GGAACTGTTACTGCAGCGAGA | 165 |
| *HIST1H2BN* | CTGGCGCATTACAACAAGC | TAATGTTCCGCGGTGGGC | 163 |
| *HIST1H2BO* | CTGGCGCATTACAACAAGC | TGCTGGCAGCTGCGAGAG | 160 |
| *NME5* | GCCAACTCTGCTTGAAGGAC | GGATGGTGACAAAGTTTGGG | 120 |
| *SMARCB1* | AGCCACTGTGGAAGAGAGGA | CTGTTCCTCTTGGCCTTCTG | 225 |
| *IGHMBP2* | GGTGGTCATTGACGAGTGTG | TCCATCAGGCTGAGTGACAG | 159 |
| *NCAPH2* | TGGATGACTTCCCTGACTCC | TTCATCCTGAAATCCTTCCG | 184 |
