# Supplementary material for: Transcription Profiling of Epstein-Barr Virus Nuclear Antigen (EBNA)-1 Expressing Cells Suggests Targeting of Chromatin Remodeling Complexes
Source: PLoS One. 2010 Aug 10;5(8):e12052. doi: 10.1371/journal.pone.0012052 (PMC2919392; doi:10.1371/journal.pone.0012052)
Supplement: Table S2 — List of genes that are regularly affected independently on the time of EBNA-1 expression. (0.08 MB DOC) [file pone.0012052.s003.doc]

**Table S2.** List of genes that are regularly affected independently on the time of EBNA-1 expression^a^.

| **Gene symbol** | **Description** | **Mean fold change^b^** | | |
| --- | --- | --- | --- | --- |
|  |  | **Short-term expression** | **Long-term expression** | **Stable expression** |
| ATP9A | ATPase, Class II, type 9A | 3.50 | 1.71 | 10.30 |
| TECT1 | Tectonic 1 | 1.57 | 2.04 | 8.54 |
| A_24_P920715 | Hypothetical protein | 5.26 | 1.31 | 4.14 |
| LGALS1 | Lectin, galactoside-binding, 1 (galectin 1) | 1.51 | 2.36 | 5.78 |
| GABARAPL3 | GABA(A) receptors associated protein like 3 | 1.43 | 1.49 | 5.75 |
| SC4MOL | C-4 methylsterol oxidase (EC 1.14.13.72) | 1.79 | 1.79 | 4.54 |
| LOC440335 | Hypothetical gene supported by BC022385 | 1.31 | 2.03 | 4.73 |
| NME5 | Non-metastatic cells 5 (nucleoside-diphosphate kinase) | 1.37 | 1.31 | 5.01 |
| EML1 | Echinoderm microtubule associated protein like 1 | 2.95 | 1.30 | 3.37 |
| CD58 | CD58 molecule | 1.57 | 1.41 | 4.18 |
| CMTM7 | CKLF-like MARVEL transmembrane domain -7 | 1.41 | 1.34 | 3.81 |
| A_32_P221305 | Hypothetical protein | 1.79 | 2.34 | 2.25 |
| TRAF1 ^c^ | TNF receptor-associated factor 1 | 1.57 | 1.35 | 2.80 |
| HIST1H2BJ ^c^ | Histone cluster 1, h2bj | 1.62 | 1.49 | 2.39 |
| TMEM2 | Transmembrane protein 2 | 2.35 | 1.42 | 1.59 |
| PROC | Protein C (inactivator of coagulation factors) | 1.67 | 1.41 | 2.20 |
| A_24_P488462 | Hypothetical protein | 1.59 | 1.40 | 2.28 |
| HIST1H2BD ^c^ | Histone cluster 1, h2bd | 1.58 | 1.60 | 1.97 |
| LOC391566 | Similar to Histone H2B 291B | 1.51 | 1.33 | 2.17 |
| HIST1H2BC ^c^ | Histone cluster 1, h2bc | 1.50 | 1.60 | 1.84 |
| HIST1H2BN ^c^ | Histone cluster 1, h2bn | 1.54 | 1.52 | 1.85 |
| HIST1H2BL ^c^ | Histone cluster 1, h2bl | 1.41 | 1.48 | 2.03 |
| HIST1H2BO ^c^ | Histone cluster 1, h2bo | 1.43 | 1.49 | 1.96 |
| HIST1H2BM ^c^ | Histone cluster 1, h2bm | 1.42 | 1.38 | 2.09 |
| MAST4 | Microtubule-associated serine/threonine-kinase 4 | 1.67 | 1.51 | 1.68 |
| HIST1H2BG ^c^ | Histone cluster 1, h2bg | 1.55 | 1.49 | 1.71 |
| ATP8B2 | Atpase, Class I, type 8B, member 2 | 1.35 | 1.26 | 2.13 |
| EFNA1 | Ephrin-A1 | 1.82 | 1.30 | 1.53 |
| ANXA2 | Annexin A2 | 1.42 | 1.27 | 1.94 |
| HIST3H2BB ^c^ | Histone cluster 3, h2bb | 1.44 | 1.53 | 1.62 |
| HIST1H2BI ^c^ | Histone cluster 1, h2bi | 1.39 | 1.44 | 1.77 |
| GTF2IRD1 | GTF2I repeat domain containing 1 | -1.51 | -1.33 | -1.41 |
| IGHMBP2 ^c^ | Immunoglobulin mu binding protein 2 | -1.30 | -1.56 | -1.41 |
| FXYD2 | FXYD domain-containing ion-transport regulator 2 | -1.53 | -1.35 | -1.44 |
| AKT2^c^ | V-akt murine thymoma viral oncogene homolog 2 | -1.33 | -1.33 | -1.72 |
| SMARCB1 | SWI/SNF related, matrix associated, actin dependent regulator of chromatin, subfamily b, member 1 | -1.43 | -1.41 | -1.60 |
| KCNH6 | Potassium voltage-gated channel, subfamily H-6 | -1.53 | -1.47 | -1.44 |
| NCAPH2 | Non-SMC condensin II complex, subunit H2 | -1.34 | -1.54 | -1.61 |
| STARD3 | START domain containing 3 | -1.48 | -1.72 | -1.42 |
| A_23_P119794 | Hypothetical protein | -1.32 | -1.35 | -2.17 |
| A_24_P7228 | Hypothetical protein | -1.46 | -1.56 | -1.59 |
| SLAMF6 | SLAM family member 6 | -1.57 | -1.32 | -1.92 |
| NAPE-PLD | N-acyl-phosphatidylethanolamine-hydrolyzing phospholipase D | -1.33 | -1.37 | -2.56 |
| GRAP | GRB2-related adaptor protein | -1.46 | -1.56 | -2.07 |
| ZBTB6 | Zinc finger and BTB domain containing 6 | -1.32 | -1.56 | -3.09 |
| BTN3A3 | Butyrophilin, subfamily 3, member A3 | -1.76 | -2.04 | -3.09 |
| MME | Membrane metallo-endopeptidase | -2.34 | -1.96 | -3.16 |

1. Genes showing at least 1.25 fold change in three independent experiments were considered as differentially regulated.
2. Mean fold change in triplicate experiments
3. Concordant changes in protein levels were observed by quantitative mass spectroscopy analysis (Ramkrishna et al. unpublished observation)
