# Supplementary material for: Transcription Profiling of Epstein-Barr Virus Nuclear Antigen (EBNA)-1 Expressing Cells Suggests Targeting of Chromatin Remodeling Complexes
Source: PLoS One. 2010 Aug 10;5(8):e12052. doi: 10.1371/journal.pone.0012052 (PMC2919392; doi:10.1371/journal.pone.0012052)
Supplement: Table S3 — List of differentially regulated genes that contain multiple putative EBNA-1 binding sites in their promoter. (0.12 MB DOC) [file pone.0012052.s004.doc]

**Table S3.** List of differentially regulated genes that contain multiple putative EBNA-1 binding sites in their promoter^a^.

| **Gene^b^** | **Description** | **Fold change** |
| --- | --- | --- |
| **Short-term expression** | | |
| ACTA1 | Actin, alpha 1, skeletal muscle | 1.39 |
| TUBB3 | Tubulin, beta 3 | -1.33 |
| PKP3 | Plakophilin 3 | -1.37 |
| SLA2 | Src-like-adaptor 2 | -1.47 |
| PHF20 | PHD finger protein 20 | -1.61 |
| **Long-term expression** | | |
| PTPRN | Protein tyrosine phosphatase, receptor type, N | 3.18 |
| EHBP1 | EH domain binding protein 1 | 1.96 |
| MAPK4 | Mitogen-activated protein kinase 4 | 1.60 |
| C6orf106 | Chromosome 6 open reading frame 106 | 1.57 |
| MAP3K6 | Mitogen-activated protein kinase kinase kinase 6 | 1.50 |
| PIGR^b^ | Polymeric immunoglobulin receptor | 1.48 |
| GNG13 | Guanine nucleotide binding protein (G protein), gamma 13 | 1.41 |
| FMO4 | Flavin containing monooxygenase 4 | 1.39 |
| TBXA2R | Thromboxane A2 receptor | 1.36 |
| TESC | Tescalcin | 1.36 |
| CAST | Calpastatin | 1.34 |
| NUDT9 | Nudix (nucleoside diphosphate linked moiety X)-type motif 9 | 1.32 |
| GRIA4 | Glutamate receptor, ionotrophic, AMPA 4 | 1.31 |
| IFIH1 | Interferon induced with helicase C domain 1 | 1.27 |
| PPP1R12A | Protein phosphatase 1, regulatory (inhibitor) subunit 12A | 1.26 |
| PHF20 | PHD finger protein 20 | -1.25 |
| ADI1 | Acireductone dioxygenase 1 | -1.27 |
| IFI30 | Interferon, gamma-inducible protein 30 | -1.27 |
| NUDT15 | Nudix (nucleoside diphosphate linked moiety X)-type motif 15 | -1.28 |
| CORO1A^b^ | Coronin, actin binding protein, 1A | -1.30 |
| SLC5A12 | Solute carrier family 5 (sodium/glucose cotransporter), member 12 | -1.30 |
| LECT1* | Leukocyte cell derived chemotaxin 1 | -1.32 |
| SEMA4A* | Sema domain, immunoglobulin domain (Ig), transmembrane domain (TM) and short cytoplasmic domain, (semaphorin) 4A | -1.32 |
| C3orf27* | Chromosome 3 open reading frame 27 | -1.32 |
| ACP1 | Acid phosphatase 1, soluble | -1.35 |
| RFT1* | RFT1 homolog (S. Cerevisiae) | -1.37 |
| EDC4 | Enhancer of mrna decapping 4 | -1.39 |
| KIAA0562 | Kiaa0562 | -1.39 |
| USP21 | Ubiquitin specific peptidase 21 | -1.39 |
| CD300C | CD300c molecule | -1.39 |
| SMYD5 | SMYD family member 5 | -1.47 |
| DLX1 | Distal-less homeobox 1 | -1.54 |
| CAMKV | Cam kinase-like vesicle-associated | -1.56 |
| SLA2 | Src-like-adaptor 2 | -1.61 |
| SLC39A3* | Solute carrier family 39 (zinc transporter), member 3 | -1.69 |
| CASP1 | Caspase 1, apoptosis-related cysteine peptidase (interleukin 1, beta, convertase) | -1.89 |
| TUBB3 | Tubulin, beta 3 | -1.96 |
| **Stable expression** | | |
| SYNPO | Synaptopodin | 9.81 |
| SLC5A12 | Solute carrier family 5 (sodium/glucose cotransporter), member 12 | 8.44 |
| CAMKV | Cam kinase-like vesicle-associated | 8.08 |
| ADI1 | Acireductone dioxygenase 1 | 3.78 |
| EPS8L2 | EPS8-like 2 | 3.74 |
| CPVL*^b^ | Carboxypeptidase, vitellogenic-like | 3.46 |
| PAPSS2 | 3'-phosphoadenosine 5'-phosphosulfate synthase 2 | 3.14 |
| FMO4 | Flavin containing monooxygenase 4 | 3.11 |
| TNNC2 | Troponin C type 2 (fast) | 2.86 |
| C3orf33 | Chromosome 3 open reading frame 33 | 2.51 |
| PIK3IP1 | Phosphoinositide-3-kinase interacting protein 1 | 2.49 |
| KCNH2 | Potassium voltage-gated channel, subfamily H (eag-related), member 2 | 2.24 |
| XRN2 | 5'-3' exoribonuclease 2 | 2.24 |
| MAP3K6 | Mitogen-activated protein kinase kinase kinase 6 | 2.15 |
| UNC13B | Unc-13 homolog B (C. Elegans) | 2.07 |
| IGF2 | Insulin-like growth factor II Precursor (IGF-II) (Somatomedin-A)[Insulin-like growth factor II Ala-25 Del] [Source:uniprotkb/Swiss-Prot;Acc:P01344] | 2.04 |
| CWF19L2 | CWF19-like 2, cell cycle control (S. Pombe) | 1.98 |
| KIN | KIN, antigenic determinant of reca protein homolog (mouse) | 1.89 |
| IFIH1 | Interferon induced with helicase C domain 1 | 1.80 |
| SLC24A6 | Solute carrier family 24 (sodium/potassium/calcium exchanger), member 6 | 1.77 |
| ST3GAL2 | ST3 beta-galactoside alpha-2,3-sialyltransferase 2 | 1.72 |
| ALS2 | Amyotrophic lateral sclerosis 2 (juvenile) | 1.62 |
| MTM1* | Myotubularin 1 | 1.57 |
| IFI30 | Interferon, gamma-inducible protein 30 | 1.46 |
| CAST | Calpastatin | 1.39 |
| NUDCD3 | Nudc domain containing 3 | 1.39 |
| ABBA-1 | Actin-bundling protein with BAIAP2 homology | 1.39 |
| IFT172 | Intraflagellar transport 172 homolog (Chlamydomonas) | 1.38 |
| SLCO4A1 | Solute carrier organic anion transporter family, member 4A1 | -1.32 |
| CRAT | Carnitine acetyltransferase | -1.35 |
| TUBB3 | Tubulin, beta 3 | -1.35 |
| SEMA4A* | Sema domain, immunoglobulin domain (Ig), transmembrane domain (TM) and short cytoplasmic domain, (semaphorin) 4A | -1.37 |
| MARK2 | MAP/microtubule affinity-regulating kinase 2 | -1.39 |
| HMGN3 | High mobility group nucleosomal binding domain 3 | -1.41 |
| ASXL1 | Additional sex combs like 1 (Drosophila) | -1.41 |
| PKP3 | Plakophilin 3 | -1.45 |
| C1orf26 | Chromosome 1 open reading frame 26 | -1.47 |
| GMIP | GEM interacting protein | -1.56 |
| ZNF613 | Zinc finger protein 613 | -1.64 |
| KIAA0562 | Kiaa0562 | -1.67 |
| PTPRT | Protein tyrosine phosphatase, receptor type, T | -1.69 |
| NUDT9 | Nudix (nucleoside diphosphate linked moiety X)-type motif 9 | -1.72 |
| ADCY4 | Adenylate cyclase 4 | -1.72 |
| TSPYL1* | TSPY-like 1 | -1.75 |
| CORO1A | Coronin, actin binding protein, 1A | -1.82 |
| NUDT15 | Nudix (nucleoside diphosphate linked moiety X)-type motif 15 | -1.82 |
| FAM49B* | Family with sequence similarity 49, member B | -1.85 |
| ITPKB*^b^ | Inositol 1,4,5-trisphosphate 3-kinase B | -1.92 |
| TIMM8B | Translocase of inner mitochondrial membrane 8 homolog B (yeast) | -2.08 |
| FBXW7 | F-box and WD repeat domain containing 7 | -2.38 |
| MYEOV | Myeloma overexpressed gene (in a subset of t(11;14) positive multiple myelomas) | -2.50 |
| C3orf38 | Chromosome 3 open reading frame 38 | -2.56 |
| JMJD2D | Jumonji domain containing 2D | -2.70 |
| FOSB | FBJ murine osteosarcoma viral oncogene homolog B | -3.03 |
| TCEAL6 | Transcription elongation factor A (SII)-like 6 | -25.00 |

1. Genes containing two or more binding sites are listed and genes with 3 binding sites are indicated by and asterisk (*).
2. Binding of EBNA-1 was confirmed by ChIP assays (Dresa*ng et a*l., 2009)
