# Supplementary material for: Transcription Profiling of Epstein-Barr Virus Nuclear Antigen (EBNA)-1 Expressing Cells Suggests Targeting of Chromatin Remodeling Complexes
Source: PLoS One. 2010 Aug 10;5(8):e12052. doi: 10.1371/journal.pone.0012052 (PMC2919392; doi:10.1371/journal.pone.0012052)
Supplement: Table S4 — List of differentially regulated TRs and contain EBNA-1 binding sites in their promoter. (0.07 MB DOC) [file pone.0012052.s005.doc]

**Table S4.** List of differentially regulated TRs and contain EBNA-1 binding sites in their promoter.

| **Gene** | **Description** | **Fold change** |
| --- | --- | --- |
| **Short-term expression** | | |
| STAT4 | Signal transducer and activator of transcription 4 | 2.23 |
| EGR2 | Early growth response 2 | 1.78 |
| TCF25 | Transcription factor 25 | -1.41 |
| ESR2 | Estrogen receptor 2 | -1.79 |
| **Long-term expression** | | |
| SATB1 | SATB homeobox 1 | 2.08 |
| TCF7L2 | Transcription factor 7-like 2 (T-cell specific, HMG-box) | 1.61 |
| MAPK4 | Mitogen-activated protein kinase 4 | 1.60 |
| TGIF2LY | TGFB-induced factor homeobox 2-like, Y-linked | 1.53 |
| AFF4 | AF4/FMR2 family, member 4 | 1.51 |
| MTF1 | Metal-regulatory transcription factor 1 | 1.51 |
| ZNF197 | Zinc finger protein 197 | 1.40 |
| LMO7 | LIM domain 7 | 1.33 |
| ARID4A | AT rich interactive domain 4A (RBP1-like) | 1.30 |
| RERE | Arginine-glutamic acid dipeptide (RE) repeats | 1.27 |
| BACH1 | BTB and CNC homology 1, basic leucine zipper transcription factor 1 | 1.27 |
| BHLHB2 | Basic helix-loop-helix domain containing, class B, 2 | 1.26 |
| VHL | Von Hippel-Lindau tumor suppressor | -1.27 |
| CLDN12 | Claudin 12 | -1.32 |
| HMGA1 | High mobility group AT-hook 1 | -1.33 |
| AP3S2 | Adaptor-related protein complex 3, sigma 2 subunit | -1.35 |
| LZTR1 | Leucine-zipper-like transcription regulator 1 | -1.52 |
| DLX1 | Distal-less homeobox 1 | -1.54 |
| RUNX1T1 | Runt-related transcription factor 1; translocated to, 1 (cyclin D-related) | -1.67 |
| **Stable expression** | | |
| CREB5 | CAMP responsive element binding protein 5 | 19.68 |
| RXRA | Retinoid X receptor, alpha | 4.98 |
| NFE2L3 | Nuclear factor (erythroid-derived 2)-like 3 | 3.48 |
| TSC22D3 | TSC22 domain family, member 3 | 2.67 |
| ARID5B | AT rich interactive domain 5B (MRF1-like) | 2.48 |
| SHOX2 | Short stature homeobox 2 | 2.34 |
| ESR2 | Estrogen receptor 2 (ER beta) | 2.27 |
| CTNNB1 | Catenin (cadherin-associated protein), beta 1, 88kDa | 2.14 |
| BACH1 | BTB and CNC homology 1, basic leucine zipper transcription factor 1 | 2.07 |
| ETS2 | V-ets erythroblastosis virus E26 oncogene homolog 2 | 1.84 |
| PBX3 | Pre-B-cell leukemia homeobox 3 | 1.59 |
| ZSCAN20 | Zinc finger and SCAN domain containing 20 | 1.54 |
| AP3S2 | Adaptor-related protein complex 3, sigma 2 subunit | 1.37 |
| TP73 | Tumor protein p73 | -1.39 |
| GTF2IRD1 | GTF2I repeat domain containing 1 | -1.41 |
| ZHX3 | Zinc fingers and homeoboxes 3 | -1.41 |
| TFDP3 | Transcription factor Dp family, member 3 | -1.69 |
| CBFA2T3 | Core-binding factor, runt domain, alpha subunit 2; translocated to, 3 | -1.96 |
| MYO1C^a^ | Myosin IC | -2.44 |
| FOSB | FBJ murine osteosarcoma viral oncogene homolog B | -3.03 |
| HOXA7 | Homeobox A7 | -16.67 |

a . TRs that contains more than one binding site for EBNA-1 in the promoter sequence
