# Supplementary material for: Transcription Profiling of Epstein-Barr Virus Nuclear Antigen (EBNA)-1 Expressing Cells Suggests Targeting of Chromatin Remodeling Complexes
Source: PLoS One. 2010 Aug 10;5(8):e12052. doi: 10.1371/journal.pone.0012052 (PMC2919392; doi:10.1371/journal.pone.0012052)
Supplement: Table S5 — List of regulated genes included in protein interaction analysis. (0.08 MB DOC) [file pone.0012052.s006.doc]

**Table S5.** List of regulated genes included in protein interaction analysis^a^.

| **Gene** | **Description** | **Mean fold change^b^** | |
| --- | --- | --- | --- |
|  |  | **Long-term expression** | **Stable expression** |
| BMI1 | BMI1 polycomb ring finger oncogene | 1.54 | 9.5 |
| VCX | Variable charge, X-linked | 1.36 | 6.82 |
| TCF7L2 | Transcription factor 7-like 2 (T-cell specific, HMG-box) | 1.61 | 6.67 |
| CBX7 | Chromobox homolog 7 | 1.59 | 3.2 |
| RSF1 | Remodeling and spacing factor 1 | 1.73 | 2.74 |
| HIST1H2AC | Histone cluster 1, h2ac | 2.29 | 2.49 |
| HIST1H2BJ | Histone cluster 1, h2bj | 1.49 | 2.39 |
| ERCC4 | Excision repair cross-complementing rodent repair deficiency, complementation group 4 | 1.29 | 2.28 |
| HIST1H2BM | Histone cluster 1, h2bm | 1.38 | 2.09 |
| C11orf30 | Chromosome 11 open reading frame 30 | 1.28 | 2.07 |
| HIST1H2BH | Histone cluster 1, h2bh | 1.48 | 2.04 |
| HIST1H2BL | Histone cluster 1, h2bl | 1.48 | 2.03 |
| HIST2H2BE | Histone cluster 2, h2be | 2.37 | 2.01 |
| HIST1H2BE | Histone cluster 1, h2be | 1.47 | 2 |
| HIST1H2BD | Histone cluster 1, h2bd | 1.6 | 1.97 |
| HIST1H2BO | Histone cluster 1, h2bo | 1.49 | 1.96 |
| TNKS | Tankyrase, TRF1-interacting ankyrin-related ADP-ribose polymerase | 1.42 | 1.89 |
| NCOR1 | Nuclear receptor co-repressor 1 | 1.31 | 1.89 |
| HIST1H2BN | Histone cluster 1, h2bn | 1.52 | 1.85 |
| HIST1H2BC | Histone cluster 1, h2bc | 1.6 | 1.85 |
| HIST1H2BI | Histone cluster 1, h2bi | 1.44 | 1.77 |
| HIST1H2BG | Histone cluster 1, h2bg | 1.49 | 1.71 |
| TERF1 | Telomeric repeat binding factor (NIMA-interacting) 1 | 1.48 | 1.63 |
| HIST3H2BB | Histone cluster 3, h2bb | 1.53 | 1.62 |
| HUWE1 | HECT, UBA and WWE domain-containing protein 1) | 2.71 | 1.57 |
| TBL1XR1 | Transducin (beta)-like 1X-linked receptor 1 | 1.51 | 1.55 |
| RNF2 | Ring finger protein 2 | 1.33 | 1.42 |
| RBBP4 | Retinoblastoma binding protein 4 | 0.8 | 0.75 |
| CBX8 | Chromobox homolog 8 (Pc class homolog,) | 0.75 | 0.74 |
| SMC1A | Structural maintenance of chromosomes 1A | 0.77 | 0.72 |
| HMGN2 | High-mobility group nucleosomal binding domain 2 | 0.78 | 0.69 |
| DAPK3 | Death-associated protein kinase 3 | 0.75 | 0.68 |
| MBD3 | Methyl-cpg binding domain protein 3 | 0.69 | 0.68 |
| SETDB1 | SET domain, bifurcated 1 | 0.79 | 0.65 |
| SMARCB1 | SWI/SNF related, matrix associated, actin dependent regulator of chromatin, subfamily b, member 1 | 0.71 | 0.63 |
| SMARCA4 | SWI/SNF related, matrix associated, actin dependent regulator of chromatin, subfamily a, member 4 | 0.68 | 0.62 |
| EHMT2 | Euchromatic histone-lysine N-methyltransferase 2 | 0.61 | 0.59 |
| PRMT7 | Protein arginine methyltransferase 7 | 0.57 | 0.59 |
| SMARCD2 | SWI/SNF related, matrix associated, actin dependent regulator of chromatin, subfamily d, member 2 | 0.73 | 0.55 |
| CHD3 | Chromodomain helicase DNA binding protein 3 | 0.73 | 0.53 |
| MAD2L1 | MAD2 mitotic arrest deficient-like 1 (yeast) | 0.8 | 0.53 |
| HMGB2 | High mobility group protein B2 | 0.69 | 0.43 |
| MRE11A | MRE11 meiotic recombination 11 homolog A | 0.75 | 0.42 |

a. Genes with similar expression pattern in long-term and stable EBNA-1 expressing cells and enriched in the GO category “Chromatin maintenance”.
